# Herpesvirus-encoded lncRNA targets host splicing by interacting with splicing factors

**DOI:** 10.1371/journal.ppat.1014072

**Published:** 2026-03-26

**Authors:** Yuan Hong, Ritu Shekhar, Sarah McMahon, Netanya Keil, Melody Baddoo, J. Monty Watkins, Hasmik Keshishian, Caroline Stanclift, Steven A. Carr, Mathias Munschauer, James M. Burke, Erik K. Flemington, Rolf Renne

**Affiliations:** 1 Department of Molecular Genetics and Microbiology, University of Florida, Gainesville, Florida, United States of America; 2 Genetics Institute, University of Florida, Gainesville, Florida, United States of America; 3 UF Health Cancer Center Institute, University of Florida, Gainesville, Florida, United States of America; 4 Department of Pathology, Tulane University School of Medicine, New Orleans, Louisiana, United States of America; 5 Department of Molecular Medicine, The Herbert Wertheim University of Florida Scripps Institute for Biomedical Innovation and Technology, Jupiter, Florida, United States of America; 6 Department of Immunology and Microbiology, The Herbert Wertheim University of Florida Scripps Institute for Biomedical Innovation and Technology, Jupiter, Florida, United States of America; 7 Broad Institute of MIT and Harvard, Cambridge, Massachusetts, United States of America; 8 Department of Infectious Diseases, Molecular Virology, Medical Faculty Heidelberg, Heidelberg University, Germany; Brigham and Women's Hospital, UNITED STATES OF AMERICA

## Abstract

Kaposi’s sarcoma-associated herpesvirus (KSHV) encodes multiple short and long noncoding RNAs which contribute to viral latency, persistence, host gene regulation, and immune evasion. The Antisense-to-Latency Transcript (ALT) is a ~ 10 kb long noncoding RNA (lncRNA) located on the opposite strand of the major latency-associated region encoding the latency associated nuclear antigen, vCyclin, vFLIP, the Kaposin’s and 12 microRNA genes. ALT is a nuclear lncRNA that is lowly expressed during latency, but strongly upregulated during lytic replication. Using RNA antisense purification and quantitative mass spectrometry (RAP-MS) in lytically induced primary effusion lymphoma cells, we identified 51 human and 3 viral proteins that directly interact with ALT. Of these enriched proteins, 48 are splicing factors, including core and alternative splicing proteins, such as U2AF2, PTBP1/2, SRSF1/3 and MBNL1. Interaction and co-localization of ALT was confirmed with various splicing factors in ribonucleoprotein complexes. We further identified that induction of lytic replication in lymphoid and epithelial cells leads to thousands of host gene splicing changes, which are partially restored upon perturbation of ALT expression. Finally, transient knockdown of ALT strongly inhibits viral reactivation and virion production. Hence, by splicing factors interactions, ALT interferes with host gene expression. Our results uncover a novel mechanism that shifts gene expression from the host to the virus late during the viral replication cycle to efficiently produce progeny virus and potentially antagonize host immune defenses.

## Author summary

LncRNAs are > 200 nt RNAs that lack significant open reading reading frames and function mainly as regulators of gene expression, including transcription, RNA processing, translation, and epigenetic control. In this study, we have identified the role of Antisense-to-LANA transcript (ALT), a KSHV lncRNA that is highly expressed during the lytic phase of viral life cycle. Our study has revealed that ALT interacts with host splicing factors that are required for cellular gene expression. We demonstrate that ALT de-regulates global host mRNA splicing during viral replication. We have also shown that the mRNA splicing changes can be restored by inhibiting the expression of ALT. Overall, we demonstrate the role of a human gammaherpesvirus encoded lncRNA in targeting host RNA splicing thereby contributing to host shut-off during viral replication.

## Introduction

Kaposi’s sarcoma-associated herpesvirus (KSHV) is the causative agent of Kaposi’s sarcoma (KS) and several other human malignancies, including primary effusion lymphoma (PEL) and a subset of multicentric Castleman’s disease (MCD) [1]. Like other herpesviruses, KSHV establishes lifelong persistent infections in its host by transitioning between latent and lytic infection cycles. KSHV transcribes both coding and non-coding RNAs that are involved in latency and the transition from latent to lytic replication and many immune evasion processes [2–4]. For example, the robustly expressed PAN (polyadenylated nuclear RNA) in KSHV has been shown to regulate reactivation and lytic replication by recruiting epigenetic modifiers to viral genomes [5,6]. Additionally, multiple microRNAs have been shown to control latency by suppressing host transcription factors and modulating host survival pathways [7–9].

**Fig 1:**
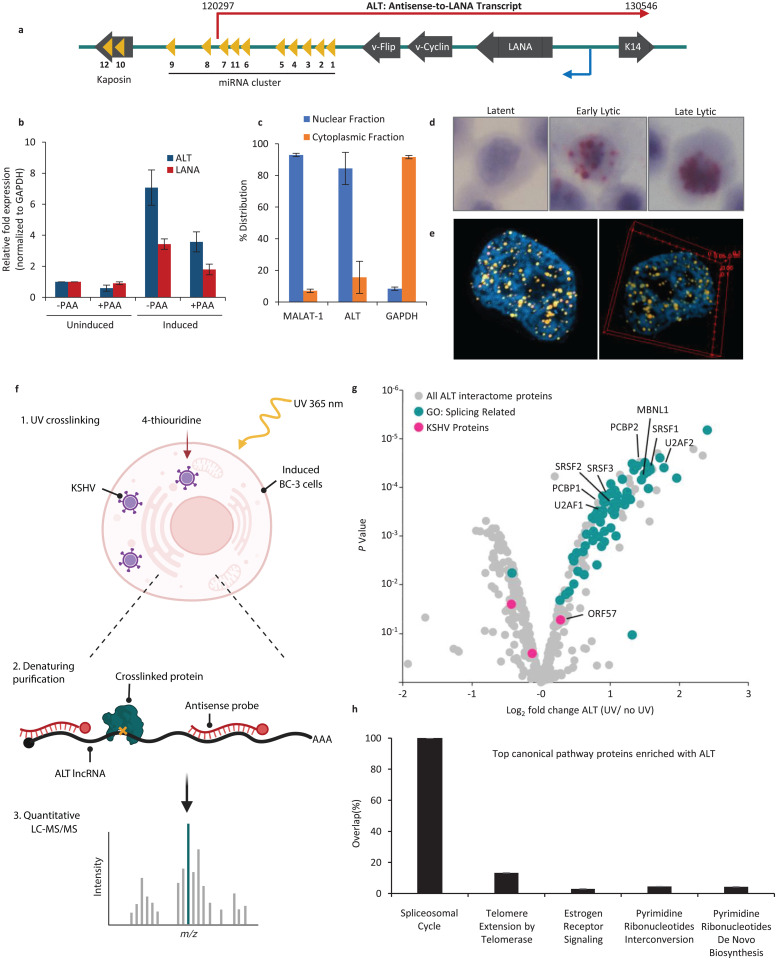
ALT transcript is nuclear localized and interacts with host splicing factors. **a)** Illustration of KSHV Latency-Associated Region (KLAR). Blue arrow is indicating the direction of latency transcripts and red arrow is indicating the direction of the ALT transcript. **b)** RT-qPCR of ALT and LANA in latent and lytic BCBL-1 cells. **c)** RT-qPCR of MALAT-1, ALT and GAPDH RNA from BCBL-1 cell cytoplasmic and nuclear fractions. **d)** RNAscope of ALT in BCBL-1 cells. **e)** 3D re-construction of ALT lncRNA in the iSLK-WT-BAC infected cell nucleus by confocal microscopy. **f)** Illustration of RAP-MS workflow on BC-3 cells under PAR-CLIP condition. Created in BioRender. Munschauer, M. (2026) https://BioRender.com/bk7ikhk **g)** Volcano plot of host and KSHV proteins enriched with ALT from RAP-MS. **h)** Ingenuity pathway analysis of ALT-binding proteins showing the top five canonical enriched pathways.

The KSHV latency-associated region (KLAR) plays a critical role in maintaining latency and contributes to pathogenesis and oncogenesis [10]. The KLAR region encodes four viral proteins including the Latency-associated Nuclear Antigen (LANA), vFLIP, vCyclin, Kaposin and 12 miRNA genes. All genes within KLAR are primarily expressed from a single major latency promotor upstream of LANA and processed by alternative splicing during latency [11] (Fig 1a). In 2014, Chandriani et al identified a > 10 kb long non-coding RNA (lncRNA) spanning nearly the entire KLAR region on the antisense strand and demonstrated that this antisense-to-LANA transcript (ALT) is induced upon reactivation from latency [12].

Dittmer and colleagues mapped the transcriptional start site (TSS) and 3’-end of ALT and identified a small intron at the 5’-end of ALT [13]. Follow-up studies applying RNA-seq approaches, including short and long read sequencing, confirmed ALT expression during lytic replication. These studies validated ALT TSS and demonstrated that ALT co-terminates with the 3’-end of K14 [3,14]. However, nothing is known about the molecular function of ALT and its role in KSHV biology. Cellular and viral lncRNAs have been shown to function in many biological processes both in the nucleus and the cytoplasm. For example, lncRNAs function as scaffolds for ribonucleoproteins (RNPs) regulating transcription, and post-transcriptional regulation, including splicing, among various cellular processes [15,16]. We aimed to unveil the function of ALT by identifying ALT-interacting proteins from lytically induced PEL cells using a stringent RNA antisense purification (RAP), coupled with quantitative liquid chromatography-mass spectrometry (RAP-MS) [17]. This analysis revealed ALT interactions with host splicing factors establishing a novel mechanism to support host shut-off during late lytic replication.

## Results

### ALT is nuclear, induced early during reactivation, and accumulates at late stages

To determine ALT expression in latent and lytic conditions, we performed RT-qPCR in uninduced and 36-h induced BCBL-1 cells. BCBL-1 is a patient-derived PEL cell line that is latently infected with KSHV and contains ~70 episomes per cell [18,19]. ALT is detectable at low levels during latency, but highly expressed upon reactivation in the presence or absence of viral DNA replication inhibitor (PAA) demonstrating that ALT expression starts at early stages and accumulates throughout the lytic replication cycle (Fig 1b). Next, we determined the cellular localization of ALT by RT-qPCR on cell-fractionated RNAs demonstrating that ALT like MALAT1, a cellular lncRNA, is predominantly localized to the nucleus (Fig 1c). In contrast, the major distribution of the GAPDH mRNA was found in the cytoplasmic fraction, as expected for mRNAs (Fig 1c). To validate these results, we performed RNAscope *in situ* hybridization for ALT in BCBL-1 and iSLK-WT-BAC cells during latency and different times post induction. ALT was not detectable during latency (Fig 1d). In congruence with the RT-qPCR data, RNAscope showed that ALT is predominantly expressed within the nucleus accumulating throughout reactivation (Figs 1d and S1). During the late lytic cycle, ALT formed large nuclear foci suggesting that ALT may form large RNP complexes visible as distinct nuclear domains (Figs 1d and S1). We further performed ALT RNAscope in induced iSLK-WT-BAC cells to study its nuclear localization at higher resolution using confocal microscopy. In contrast to large foci observed by light microscopy in BCBL-1, we observed more discrete nuclear punctate signals in iSLK-WT-BAC cells (Fig 1e). Together, these data show that ALT is predominantly expressed in the nucleus and resides in punctate structures, suggesting ALT forms RNP complexes of unknown composition.

### Identification of ALT interacting proteins

To elucidate ALT function, we identified the ALT RNA-protein interactome using RAP-MS that combines protein denaturing purification conditions with native UV-crosslinking to identify direct interactions between RNA and proteins in intact cells. Specifically, we implemented a photoactivated ribonucleoside-enhanced crosslinking procedure, derived from PAR-CLIP, to BC-3 cells, another PEL cell line latently infected with KSHV which efficiently uptakes 4-thiouridine (4-sU) (Fig 1f) [17]. As a control, ALT was purified from non-crosslinked cells. We performed two replicate experiments and analyzed the protein content in each RNA purification using Isobaric Tags for Relative and Absolute Quantitation (iTRAQ)-based quantitative mass spectrometry. We detected 434 proteins with at least two unique peptides. We identified 50 host proteins and a KSHV RNA binding protein, ORF57, that were at least two fold enriched relative to the control across all replicates and displayed statistical significance (adjusted *P* value <0.005) (Fig 1g and S1 Table). Gene ontology analysis revealed that 48 of the 50 candidate ALT-interacting proteins and ORF57 are splicing factors. Ingenuity pathway analysis of the potential ALT interactors (adjusted *P* < 0.005) showed the enrichment of splicing, telomere extension and pyrimidine ribonucleotide interconversion and biosynthesis pathways among the top five canonical pathways, with splicing being the highest ranked pathway (Fig 1h).

### ALT interacts with multiple host-splicing factors

**Fig 2:**
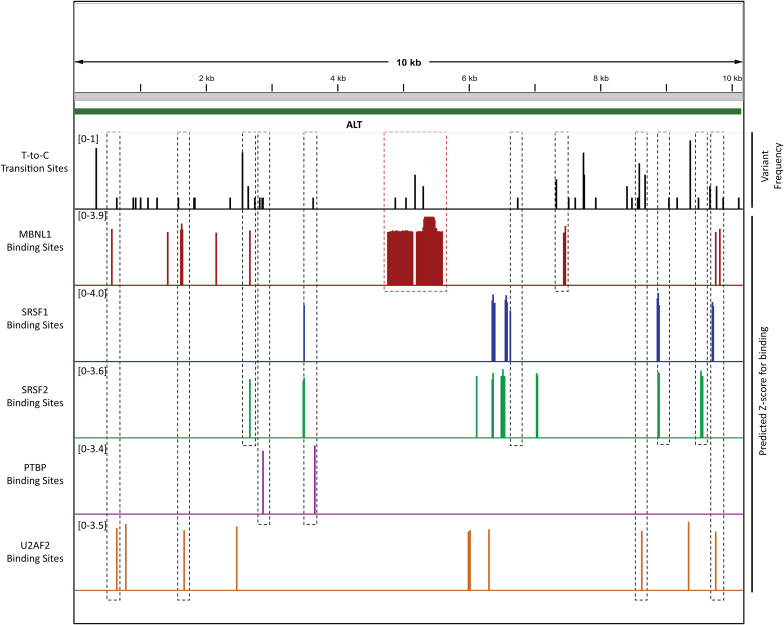
T-to-C transition sites and predicted protein binding sites on ALT. The IGV panel shows the complete ALT sequence (130696-120545 on the NC_009333.1 reference genome) with T-to-C substitution sites (black bars) identified on the RAP-purified ALT RNA. The length of bars represent the variant frequency. All other panels depict the binding sites (Z-score >3, p < 0.05) for various splicing factors including MBNL1 (red), SRSF1 (blue), SRSF2 (green), PTBP (purple) and U2AF2 (orange). The dotted blocks encompass the predicted protein binding sites which are most proximal (<150 bp) to the identified T-to-C transition sites. The red dotted block encompass all the predicted MBNL1 binding sites in the ALT repeat region.

Enrichment of both positive and negative splicing regulators with ALT suggested that the abundant expression of ALT may perturb alternative splicing patterns during lytic replication. These proteins included muscleblind-like protein 1 (MBNL1), polypyrimidine tract binding proteins (PTBP1/2), U2 auxilary factor proteins (U2AF), serine/arginine-rich proteins (SRSF1/2/3 and ASF/SF2) and heterogenous nuclear ribonucleoproteins (HNRNPs). To validate that ALT directly interacts with these splicing factors, we sequenced ALT RNA purified from RAP and identified T-to-C transitions in the RNA caused by UV-crosslinking under PAR-CLIP conditions. Mapped T-to-C transition sites represent protein binding motifs on ALT (S2 Table). Next, we interrogated the ALT sequence for RNA binding motifs of well-characterized splicing factors using RBPmap [20]. We found that the ALT sequence contains multiple binding sites with Z-score > 3 and *P* < 0.05, considered as highly significant, for the analyzed splicing factors (S2 Table). We observed that the cognate binding sites for MBNL1, PTBP1/2 and U2AF1/2 are in close proximity (150 bp) of T-to-C transitions in the ALT RNA, providing additional evidence for direct ALT splicing factor interactions (Fig 2). Of note, the strongest binding sites for MBNL1 are predicted within the repeat region on ALT, reminiscent to expanded CUG and CCUG repeats, on DMPK and ZNF9 mRNAs, which sequester MBNL1 in muscular dystrophy [21,22].

### ALT co-localizes with splicing factors in nuclear ribonucleoprotein complexes

**Fig 3:**
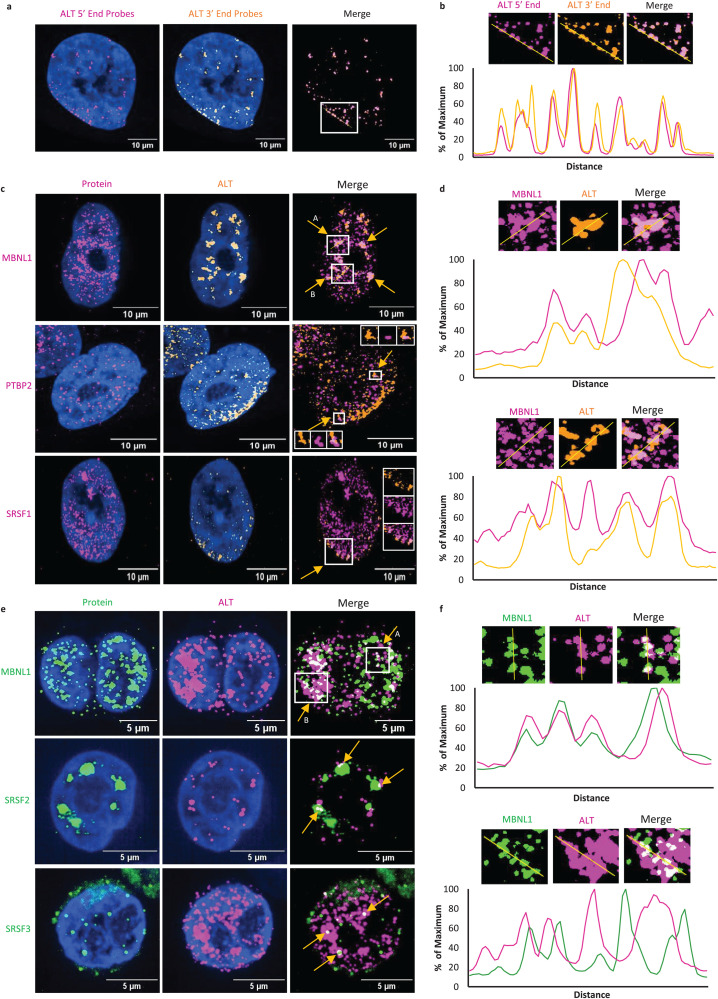
Colocalization of ALT with host splicing factors. **a)** smFISH for ALT using probes targeting 5’-end (magenta) or the 3’-end (yellow) of the ALT RNA in iSLK-WT-BAC16 infected cells. **b)** Magnified signals for the region marked by white box in Fig 3a. **c)** IFA detecting different cellular proteins including MBNL1, PTBP2 and SRSF1 (magenta) combined with smFISH-ALT (yellow) in iSLK-WT-BAC16 infected cells. Colocalization of the two signals (pink) is shown in the merged images, with insets in PTBP2 and SRSF3 showing the magnified signals for the marked regions **d)** Magnified signals of ALT and MBNL1 for regions marked in MBNL1 merge image (Fig 3c) with arrows A (top) and B (bottom). The plots display the intensity of signals across the yellow line. **e)** IFA detecting different cellular proteins including MBNL1, SRSF2 and SRSF3 (green) combined with smFISH-ALT (magenta) in BC-3 cells. Colocalization of the two signals (white) is shown in the merged images. **f)** Magnified signals of ALT and MBNL1 for regions marked in MBNL1 merge image (Fig 3e) with arrows A (top) and B (bottom). The plots display the intensity of signals across the yellow line. Nucleus is stained with DAPI (blue) in all images.

To analyze co-localization of ALT with splicing factors, we used different fluorescence microscopy techniques in KSHV-infected cells. First, we performed single molecule fluorescence in situ hybridization (smFISH) to detect ALT in lytic reactivated iSLK-WT-BAC infected cells. To establish that the smFISH probes detect complete ALT RNA, we performed smFISH with two different sets of probes, one set targeting the 5′-end and the other targeting the 3′-end of ALT. We observed nearly complete co-localization between the 5′-end and 3′-end probes, establishing that both probes detect ALT (Fig 3a and 3b). Next, we combined smFISH with immunofluorescence (IF) to detect proteins of interest (Fig 3c–3f). We found that ALT co-localizes with MBNL1, PTBP2 and SRSF1 in iSLK-WT-BAC infected lytic cells (Fig 3c). The observed degrees of co-localization and signal intensity varied between ALT and the respective splicing factors. For example, PTBP2 forms small speckles that co-localize with ALT mainly in the periphery of the nucleus, while MBNL1 forms large foci with ALT, with high degree of co-localization determined by overlapping signal intensity peaks for the two signals (Figs 3d and S2). The Spearman’s rank correlation coefficient of ALT colocalization with each splicing factor in induced iSLK-WT-BAC cells was estimated as 0.64 for MBNL1, 0.6 for SRSF1 and 0.24 for PTBP2 (S2 Fig). Similar results were observed in lytic BC-3 cells (Fig 3e). ALT co-localized with MBNL1, SRSF2 (Sc-35) and SRSF3, with highest colocalization of ALT observed with MBNL1 (Fig 3f). The Spearman’s rank correlation coefficient of ALT colocalization with each splicing factor in the induced BC-3 cells was estimated as 0.54 for MBNL1, 0.56 for SRSF3 and 0.27 for SRSF2. Furthermore, RNAsocpe for ALT combined with IF for PTBP2 and SRSF1 also revealed co-localization of these proteins with ALT in iSLK cells (S3 Fig). The co-localization between ALT and splicing factors confirmed our RAP and binding motif analyses showing that ALT directly interacts with these splicing factors.

### Host splicing is altered during KSHV lytic induction

Given the strong association of ALT with splicing factors we next examined any alterations to host splicing patterns. We performed RNA-seq analysis in lytic and latent TREx-BCBL1-Rta cells [23] and compared RNA splicing between these samples using rMATS, which detects and quantifies the difference in the inclusion level of five major classes of alternative splicing events: skipped exon (SE), retained intron (RI),alternative 3’ splice site (A3SS), alternative 5’ splice site (A5SS) and mutual exclusive exon (MXE) [24].

**Fig 4:**
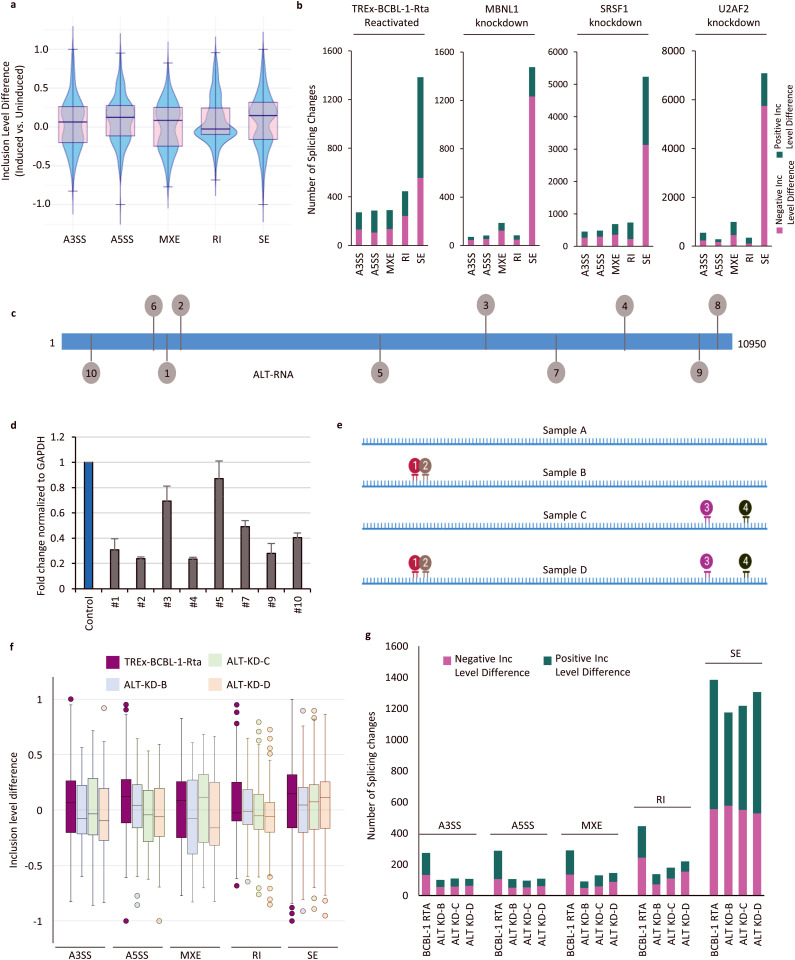
Host alternative splicing pattern is substantially changed upon KSHV reactivation and ALT knockdown. **a)** Distribution of inclusion level differences in the alternative splicing events in the reactivated TREx-BCBL1-Rta cells compared to latent cells. The alternative splicing events are characterized into 5 key categories: A3SS = Alternative 3’ Splice Site; A5SS = Alternative 5’ Splice Site; MXE = Mutually Exclusive Exons; RI = Retained Intron; SE = Skipped Exon **b)** The number of alternative splicing changes quantified as positive or negative inclusion level difference in TREx-BCBL-1-Rta cells compared to latent cells, MBNL1 knockdown in K562 cells, SRSF1 knockdown or U2AF2 knockdown in HepG2 cells compared to the control cells. **c)** Illustration of target regions for 10 different GapmeRs designed to target ALT expression. **d)** ALT knockdown efficiency in BCBL-1 cells measured by qRT-PCR for ALT using primers designed in ALT specific (LANA intronic) region. **e)** Combination of GapmeRs selected for treating BCBL-1 cells. **f-g)** Distribution of inclusion level differences (f) and the total numbers (g) of the alternative splicing changes (FDR < 0.05) in the ALT knockdown (KD) BCBL-1 samples B, C, and D (as shown in 4e), compared to control sample A, after 48 h of induction with doxycycline. The first bar for each category shows the distribution (f) and numbers (g) of alternative splicing changes (FDR < 0.05) in 48 h induced TREx-BCBL-1-Rta cells compared to control cells.

Compared to latently infected cells, we observed 2,679 splicing changes in TREx-BCBL1-Rta cells reactivated for 48-h (Fig 4a). Among the five different splicing types, increased or decreased SE changes are the most observed category with 1,384 calls for 1,053 genes. We then confirmed these observations in iSLK-WT-BAC16 infected cells. In induced iSLK cells, we observed alternative splicing changes especially SE at very comparable levels to that in TREx-BCBL1-Rta cells (S4a Fig). Next, we ectopically expressed ALT in the uninfected-iSLK cells using a pcDNA-ALT plasmid and compared the difference in the mRNA splicing with control-plasmid transfected cells (S4b Fig). Interestingly, we observed that ALT overexpression in the uninfected-iSLK cells results in significant change in the inclusion of all categories of alternative splicing events (S4c Fig). This data confirms that the expression of ALT alone without KSHV infection is sufficient to dysregulate alternative splicing in the host cells.

Similar splicing changes were detected in BCBL-1 cells, albeit at lower numbers, reflecting the lower efficiency of lytic induction compared to TREx-BCBL1-Rta cells and iSLK cells. Interestingly, about 219 of the 1,384 exon skipping events (FDR < 0.05) affected MBNL1 targets as previously determined by CLIP-seq in mouse cells [25], which is in agreement with high degree of ALT-MBNL1 co-localization demonstrated by smFISH/IF (Fig 3). This data shows that during late lytic replication host cellular splicing is globally altered. To evaluate the extent and significance of these changes, we compared our data with previously published splicing analyses upon siRNA knockdown of splicing factors, MBNL1 in K562 cells and U2AF2 and SRSF1 in HepG2 cells [26,27]. We found that the numbers of splicing changes in MBNL1 knockdown K562 cells were highly comparable to that observed in reactivated PEL cells, while the core splicing factors SRSF1 and U2AF2 knockdown, induced very high numbers of alternative splicing changes (Fig 4b). More than 50% of splicing changes in each knockdown were identified as SE yielding similar patterns of alternative splicing, when compared to the induction of KSHV lytic replication, emphasizing genome-wide impact of KSHV on splicing. A direct comparison among these datasets is however limited due to the experiments performed in distinct cell types.

KSHV and other herpesviruses lytic replication is known to alter the cellular transcriptome and proteome by host shut-off mechanisms mediated by SOX (ORF37), which causes mRNA degradation in the cytoplasm, This potentially also affects the abundance of splicing factors [28]. Therefore, we tested whether the observed splicing changes are due to the interaction between ALT and host splicing factors. To determine that the effect on splicing is mediated by ALT, we performed transient knockdown of ALT using GapmeRs targeting different regions of the ALT transcript (Fig 4c and S3 Table). The efficiency of ALT knockdown was found to be highly variable for different GapmeRs (Fig 4d). Based on the knockdown efficiency, we pooled 3 different sets of GapmeRs to transfect BCBL-1 cells (Fig 4e). Knockdown efficiency between the different groups of GapmeRs ranged between 35% and 45%. After 24-h of transfecting BCBL-1 cells with either of the ALT-GapmeR pools or control GapmeRs, we induced these cells and performed RNAseq on each sample 48-h post induction. Splicing analysis was performed as described above. We observed that ALT knockdown in induced BCBL-1 cells globally perturbed the inclusion level (FDR < 0.05) in all classes of splicing events, mostly in the opposite direction, as observed earlier for reactivation (Fig 4f). In GapmeR transfected cells, the number of splicing changes for each ALT knockdown sample vs. control-transfected samples were found comparable. Moreover, knockdown of ALT reduced the number of splicing changes for all categories further supporting that alteration of host RNA splicing is mediated by ALT expression (Fig 4g).

### Perturbation of exon skipping by ALT

**Fig 5:**
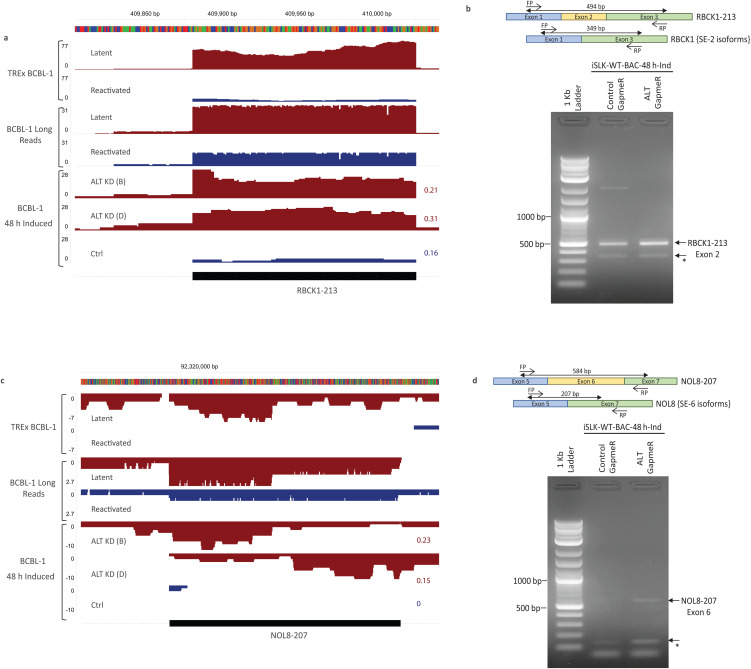
Perturbation of Exon Skipping in ALT dependent manner. **a)** An IGV plot depicting the RNAseq reads from latent and reactivated TREx-BCBL1-Rta cells, nanopore long reads from latent and reactivated BCBL-1 cells and RNAseq reads from either control-GapmeR treated or ALT-GapmeR treated BCBL-1 induced cells, spanning Exon-2 of the RBCK1-213 (black) human transcript. All reactivated and control GapmeR treated samples demonstrate the conditions with high ALT expression (blue), and all latent and ALT- GapmeR treated samples demonstrate the conditions with low ALT (red). Average normalized expression of Exon-2 of RBCK1-213 in Gapmer samples is shown on the tracks. **b)** PCR amplification of RBCK1-213 Exon 2 in control-GapmeR and ALT-GapmeR treated iSLK-WT-BAC induced cells. **c)** An IGV plot depicting the tracks for same samples as shown in 5a spanning Exon-6 of the NOL8-207 human transcript. Average normalized expression of Exon-6 of NOL8-207 in Gapmer samples is shown on the tracks. **d)** PCR amplification of NOL8-213 Exon 6 in control-GapmeR and ALT-GapmeR treated iSLK-WT-BAC induced cells. *Bottom arrows in Fig 5b and 5d mark the amplification of alternative transcript where the respective exon is skipped.

We next analyzed exon skipping changes with ALT knockdown in BCBL-1, TREx-BCBL1-Rta, and BCBL-1 reactivated samples. BCBL-1 long-read direct-cDNA sequencing, previously performed using Nanopore was used to test if long reads validate our analysis of selected gene-specific splicing changes [3]. To identify ALT-dependent specific changes, we first shortlisted SE events with significant inclusion level difference (FDR < 0.05) and selected events that showed a similar exon inclusion pattern in at least two of the three knockdown samples. Next, we tested if any of these events were identified significantly (FDR < 0.05) reversed in TREx-BCBL1-Rta reactivated samples compared to control. Interestingly, we identified that the RBCK1 and NOL8 genes showed significant changes in their exon 2 and exon 6 skipping, respectively, in an ALT-dependent manner. We observed that exon 2 in RBCK1–213 transcript remains included during latency and is skipped more in reactivated TREx-BCBL1-Rta cells with a similar pattern observed from long read sequencing of BCBL-1 cells (Figs 5a and S5a). This exon skipping in reactivated cells was found significantly reduced in ALT knockdown compared to control BCBL-1 cells (Fig 5a). We further validated the increase in exon inclusion by RT-PCR after ALT knockdown in iSLK-WT-BAC cells (Fig 5b). The NOL8–207 transcript exon 6 is another strongly significant SE event identified as dependent on ALT expression, with more skipping in conditions with high ALT expression (Figs 5c and S5b). Conversely, exon skipping in reactivated iSLK-WT-BAC cells was found to be reduced with ALT knockdown (Fig 5d). We also observed that both NOL8 and RBCK1, showed reduced expression of the skipped-exon transcript isoform as well, indicating an effect on the overall expression of these genes by host-shutoff or potential NMD of the skipped exon transcripts (Fig 5d). Based on these analysis we propose that ALT interacts with numerous splicing factors, shifting gene expression from host to the virus during late lytic replication.

### High ALT expression is required for efficient reactivation and virus production

**Fig 6:**
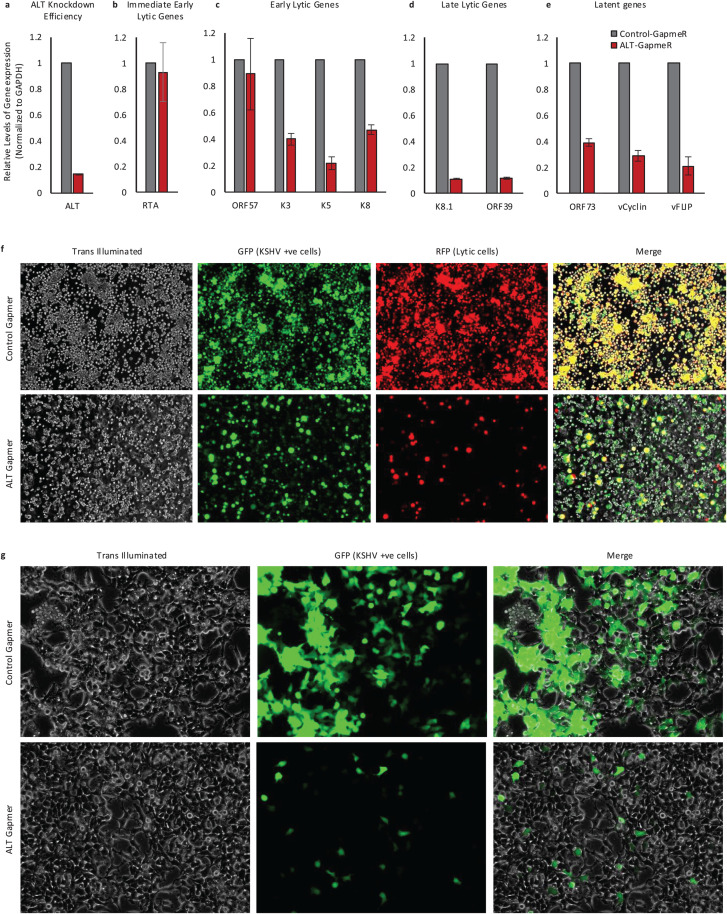
Effect of ALT knockdown on KSHV gene expression profile, reactivation and infection potential. **a-e)** The bar charts depicting the relative change in fold expression of **a)** ALT, **b)** Immediate early lytic genes including RTA, **c)** early lytic genes including ORF57, K3, K5 and K8, **d)** late lytic genes including K8.1 and ORF39 and **e)** latency associated gene LANA (ORF73), in ALT-GapmeR treated iSLK.219 cells compared to control-GapmeR treated iSLK.219 cells. **f)** Representative images of iSLK.219 cells transfected with either control-GapmeRs or ALT-GapmeRs after 72 h of induction with doxycycline. The GFP expression depicts the infected cells and the RFP expression depicts the reactivated cells. **g)** Representative images of 293T cells transduced with supernatant derived from 96 h induced control-GapmeRs or ALT-GapmeRs treated iSLK.219 cells. The GFP positive cells depict the infected cells.

Based on this model whereby ALT contributes to host shut-off by globally perturbing splicing, we wondered whether high ALT expression contributes to efficient reactivation and production of progeny virus. To test this, we transiently transfected latently infected iSLK-219-BAC cells with either control or ALT-targeting GapmeRs 24-h prior to induction for 48-h. The ALT-GapmeR transfection reduced ALT expression by more than 80% (Fig 6a). Next, we tested the effect of ALT knockdown on the expression profile of different KSHV genes characterized as immediate early, early lytic, late lytic, and latent genes using qRT-PCR (Fig 6b–6e). ALT knockdown had negligible effect on immediate early gene RTA and the early gene ORF57 (Fig 6b and 6c). The expression of early lytic genes K3, K5 and K8 was significantly reduced by more than 50%, and the highest reduction (~90%) in expression was observed for late lytic genes K8.1 and ORF39 (Fig 6c and 6d). Expression of the latency genes inclduing LANA, vFLIP and vCyclin was also reduced significantly in the ALT knockdown samples (Fig 6e). The profound reduction in the expression of both lytic and latent genes with ALT knockdown suggests a potential role of ALT in transcriptional and post-transcriptional regulation of viral gene expression as well, that will be addressed in future studies.

Next, we tested GapmerR transfected cells for GFP and RFP expression to measure reactivation. The iSLK-219-BAC cells express GFP during latency and activate RFP expression during lytic replication. While we observed an overall reduction in the GFP positive cells suggesting that ALT knockdown decreases the viral genome copies and cell viability, we observed a marked reduction in the ratio of RFP/GFP in cells transfected with ALT GapmeRs compared to control GapmeRs (Figs 6f and S6). Since in this system RFP expression is driven by an early promotor representing the reactivated cells, this data shows that ALT expression contributes to efficient reactivation from latency. To further demonstrate whether ALT expression contributes to efficient progeny virus production, we collected the supernatants from the GapmeR-transfected iSLK-219-BAC cells and transferred them to naive 293T cells and determined GFP expression. We found that the resulting infection was strongly reduced in supernatants from ALT-knockdown cells compared to control GapmeR treated cells (Fig 6g). This observation in conjunction with gene expression demonstrates that ALT expression contributes to both reactivation from latency and efficient virion production.

## Discussion

Early unbiased gene expression and transcriptomics studies revealed that vast majority of our chromosomes are expressed and tens of thousands of transcripts do not have protein coding potential. LncRNAs have arbitrarily been defined as non-coding transcripts of greater than 200 nucleotides in length [16]. Many lncRNAs are expressed in a highly cell- and tissue-specific fashion, and their altered expression patterns can be highly consequential leading to defects in development and various human diseases including cancer. For example, MALAT1, NEAT1, and GAS5 are evolutionary conserved lncRNAs that are also de-regulated in cancers [29,30]. The first viral non-coding RNAs described were herpesvirus encoded lncRNAs EBER1 and EBER2 of Epstein Barr Virus (EBV) that are highly expressed in EBV-associated cancers [31]. Recent high-resolution analysis of herpesvirus transcriptomes has identified many more lncRNAs in alpha-, beta-, and gamma-herpesviruses [3,32–34]. LncRNAs have demonstrated a multitude of functions, which makes studying their role in host and viral biology challenging. The most common interaction-dependent mechanisms for lncRNAs, are RNA/chromatin, RNA/Protein, and RNA/RNA interactions [29]. A major challenge for all lncRNA interaction studies is distinguishing physiologically occurring interactions from background noise, especially for large RNAs [35,36]. In this study, we aimed to define the role for a previously detected yet functionally uncharacterized KSHV lncRNA, ALT.

Performing RAP-MS for ALT identified that proteins bound to ALT during the viral lytic cycle were enriched with host proteins involved in mRNA-splicing pathways. These interactions were verified by motif binding analyses and high-resolution imaging of RNA-protein colocalization in cells. We found a robust increase in host splicing changes during reactivation and under ALT knockdown conditions, suggesting that ALT sequesters splicing proteins to regulate host gene expression. Interestingly, the majority of these alternative splicing changes were skipped exons, which statistically can lead to nonsense-mediated decay (NMD) of mRNAs in about 35% of the events [37,38]. Additionally, ALT knockdown in epithelial cells demonstrated that expression of this viral lncRNA is essential for efficient viral lytic replication and infection.

Our studies suggest a model by which ALT interaction with splicing factors constitutes a novel host shutoff mechanism for KSHV. We note that regulation of splicing has been reported for other nuclear lncRNAs [39]. NEAT1 and MALAT1 affect the distribution of splicing factors in nuclear speckles [30,40,41]. For example, MALAT1 modulates the speckle association of some SR proteins like SRSF1 and SRSF3 thereby regulating alternative splicing [41]. With respect to the KSHV host shutoff mechanism, an ORF37 encoded protein, also termed shutoff and exonuclease (KSHV SOX), is the only known major key player that regulates this process [42]. SOX induces the cleavage of host mRNAs within the cytoplasm thereby inducing global host shutoff [43]. While SOX can induce cleavage of many host RNAs during lytic replication, ectopic expression of SOX in uninfected cells does not recapitulate the extent observed in infected cells [44]. Additionally, SOX also has a DNase activity required for resolution of genome replication intermediates, which is both temporarily and spatially separated [45]. Specifically, SOX-dependent host RNA degradation occurs early during reactivation in the cytoplasm while genome processing takes place later in the nucleus. Given the accumulation of ALT in later stages of lytic replication, we propose that the ALT-dependent inhibition of mRNA-splicing adds a novel layer of inhibiting host gene expression when SOX is predominantly localized in the nucleus for viral DNA processing. Our finding that inhibition of ALT strongly reduced late viral gene expression further supports this model. We note that most KSHV structural proteins expressed during late lytic phase are intronless, and hence do not require splicing. Interestingly, we also observed a strong interaction of ALT with the viral post-transcriptional regulator, ORF57 that regulates the splicing of viral lytic pre-mRNAs [46]. ORF57 has also been reported to interact with host pre-mRNAs in the nucleus, thereby extending their half-life time and nuclear retention [47]. It will be interesting in future studies to determine whether this splicing-independent ORF57 activity is mediated by its interaction with ALT.

Overall, knowing that SOX-mediated degradation is based on specific secondary structure of RNAs and ALT potentially targets various host-regulatory pathways by induction or inhibition of specific alternative splice variants has lead us to hypothesize that KSHV utilizes more than one mechanism for host shutoff during its lytic replication [43]. This hypothesis is supported by the fact that nearly 20% of the total 1,053 genes which showed significant SE in reactivated PEL cells, are previously reported as MBNL1-dependent SE targets [25]. However, gene ontology analysis of these genes did not reveal any significantly enriched pathways. Studying potential roles of the MBNL1 or other enriched alternative splicing factor target genes, currently beyond the scope of this research, requires additional studies like Ribo-seq and host cell proteomics analysis.

In summary, we propose a novel host shutoff mechanism using a highly expressed lncRNA potentially by sequestering splicing factors, thereby further shifting gene expression from host to viral genes. The observation that knockdown of ALT strongly abrogates virion production points to a potentially novel antiviral strategy by RNA targeting which could address the resistance issues of current antivirals.

## Materials and methods

### Cell culture and lytic reactivation of infected cells

BC-3, BCBL-1, and TREx-BCBL1-Rta were cultured in RPMI 1640 media supplemented with 10% fetal bovine serum (FBS), 2 mM glutamine, 1 mM sodium pyruvate, 100 U/ml penicillin and 100 mg/ml streptomycin at 37°C under 5% CO2. iSLK cells [48] were cultured in Dulbecco’s modified Eagle medium (DMEM) supplemented with 10% FBS, 100 U/ml penicillin and 100 µg/ml streptomycin, 1 µg/ml puromycin, 250 µg/ml G418 and 250 µg/ml hygromycin at 37°C under 5% CO2. Lytic reactivation in BC-3 and BCBL-1 cells was induced with 12-O-tetradecanoylphorbol-13-acetate (TPA) (20 ng/ml) and sodium butyrate (2 mM), while lytic reactivation in iSLK cells and TREx-BCBL1-Rta cells was induced by using a combination of doxycycline (1 µg/ml) and sodium butyrate (1 mM). To inhibit viral DNA replication, phosphonoacetic acid (PAA) was added along with induction reagents.

### Quantitative reverse transcription-PCR (RT-qPCR) analysis

RNA was extracted from uninduced and induced PEL cells or iSLK cells using RNAbee (Tel-Test, Friendswood, TX) according to manufacturer’s protocol. Total RNA was quantified using Nanodrop 1000 spectrophotometer (Thermo Scientific, Waltham, MA), and 1 μg RNA was Turbo DNase treated (Invitrogen, Carlsbad, CA), followed by cDNA preparation with SuperScript IV First-Strand Synthesis System Kit (ThermoFisher). All quantitative RT-PCR (qRT-PCR) were performed on Roche LightCycler 96 and relative quantification was calculated by normalizing the expression of gene of interest to the house keeping Glyceraldehyde 3-phosphate dehydrogenase (GAPDH) gene expression. Student t-tests were performed for statistical significance.

### RNA antisense purification and protein elution

RAP-MS was performed as previously described [17]. Briefly, to capture endogenous ALT transcripts, we designed and synthesized 5′ biotinylated 90-mer DNA oligonucleotides (Integrated DNA Technologies) antisense to the complementary target RNA sequence. We used 96 probes such that one probe binding site occurred roughly every 400 bases in the full-length ALT lncRNA, excluding regions that matched to human transcripts or genomic regions. For the ALT RNA antisense purifications, 800 million BC-3 cells were used per replicate. Cells were induced with 20 ng/ml TPA and 2 mM sodium butyrate for 36 h followed by labelling with 200 μM 4-thiouridine (4sU) for 18 h. Cells were washed once with PBS and then crosslinked on ice using 0.8 J/cm^2^ of 365 nm UV light in a GS Gene Linker (Bio-Rad Laboratories). We generated two biological replicates from UV-crosslinked and two biological replicates from non-crosslinked cells. Preparation of cell lysates, RAP and protein elution was carried out as described previously [49]. A fraction (~10%) of each hybridized lysate, was aliquoted separately for RNA extraction to analyse T-to-C transitions from the purified ALT transcripts by RNA sequencing.

### RAP–MS protein digestion and iTRAQ labelling

RAP-captured proteins were resuspended in 40 μl of 8 M of urea in 50 mM of Tris-HCl., 4 mM of dithiothreitol (DTT) was added for 30 min at room temperature and alkylation (1.6 μl of 250 mM IAA, 45 min, dark, room temperature). All samples were then digested with Lys-c (0.1 μg per sample) for 2 h at room temperature and urea concentration was diluted to <2 M with 120 μl of 100 mM Tris-HCl pH 7.8. Next, 0.5 μg of trypsin was added to each sample for overnight digestion at room temperature followed by quenching with 8.5 μl of formic acid and desalting on 4-punch STAGE-Tips as previously described [50]. Labelling of desalted peptides was performed according to the manufacturer’s instructions (AB Sciex) with iTRAQ4 reagent [51]. Briefly, peptidesdissolved in 30 μl of 50 mM triethylamonium bicarbonate (TEAB) pH 8.5 were incubated with labelling reagent (in 70 μl of ethanol)for 1 h with agitation.Reaction was quenched with 5 μl of 1 M Tris-HCl pH 7.8. Differentially labelled peptides were subsequently mixed and prepared for BRP fractionation on 50 mg SepPak columns.Cartridges were prepared for desalting by equilibrating with methanol, 50% acetonitrile (ACN), 1% formic acid and 3 washes with 0.1% TFA and then samples were loaded on to the cartridge and washed 3 times with 1% formic acid. A pH switch was performed before the collection of BRP fractions with 5 mM ammonium formate at pH 10. BRP fractions were collected at the following ACN concentrations (all in 5 mM ammonium formate): 10% ACN; 15% ACN; 30% ACN; 40% ACN; and 50% ACN.

### RNA isolation from RAP lysate fraction and sequencing

RNA isolation was performed as described previously [52]. Briefly, beads were resuspended in 20 μl NLS RNA Elution Buffer (20 mM Tris pH 8.0, 10 mM EDTA, 2% NLS, 2.5 mM TCEP). To release the target RNA, beads were heated for 2 min at 95 °C. Beads were then magnetically separated and the supernatants containing eluted target RNA were digested by the addition of 1 mg ml − 1 Proteinase K for 1 h at 55 °C to remove all proteins. The remaining nucleic acids were then purified by ethanol precipitation onto SILANE beads (Invitrogen) as previously described 13,34. DNA probes were removed by digestion with TURBO DNase (Ambion). To quantify RNA yield and enrichment, qPCR was performed before sequencing. Sequencing was performed on an Illumina platform NovaSeq 6000 to generate 150 bp paired-end reads. Raw sequence data were processed following the standard RNA-Seq bioinformatics pipelines, including adapter trimming, quality filtering, and alignment to the reference genome (NC_009333.1) using STAR aligner.

### Quantification of 4-SU incorporation and binding motif sequence prediction

To evaluate RNP binding sites, the reads aligned to ALT transcript was compared between cross-linked and non-cross-linked samples. The majority of RNA reads from cross-linked samples displays a T-to-C conversion near or above 50%. Clusters counts of the base conversion in cross-linked samples minus noncorsslinked samples are calculated as the mutation percentage. RBPmap web tool was used to predict RNA-binding protein (RBP) binding sites on ALT sequence based on known motif information. Z-score and *P*-value are the statistical measures determined by RBPmap that predict the binding potential of each RBP including MBNL1, PTBP, SRSF1/2 and U2AF with ALT. The sites predicted with high Z-score [> 3] and low P-value (*P* < 0.05) were analysed further for their proximity (within 150 bp) to the T-to-C transition sites and were considered as robust putative RNA binding sites.

### smFISH and immunofluorescence staining for colocalization

To dual stain for immunofluorescence and smFISH, glass coverslips were pre-coated with poly-lysine and one million cells were adhered per coverslip. Cells were then fixed with 4% paraformaldehyde for 15 min followed by permeabilization with 70% ethanol at 4°C overnight. Cells were washed twice with nuclease free PBS and then incubated in 500 μl of primary antibody (SRSF1; Invitrogen #32–4500 1:250, SRSF3; Invitrogen #32–4200 1:250, SRSF2/Sc-35; Abcam #Ab11826 1:250, PTBP1; Invitrogen #32–4800 1:250, PTBP2; Sigma #HPA047420 1:100, MBNL1; Abcam #Ab45899 1:1000) in PBS for 4 h at 4°C. Cells were washed twice in PBS, then secondary antibodies tagged with either Alexa-Fluor 555 or Alexa-Fluor 647 (Invitrogen 1:1000), were added for 2 h at 4°C. Cells were washed twice, then fixed in 4% PFA (Fisher Scientific Co LLC: 50980495) for 10 min. Cells were washed three times in PBS, then washed in buffer A (filter-sterilized 2x SSC with 10% formamide) for 5 min. smFISH probes were then added to a hybridization chamber (square Petri dish cat #FB0875711A) containing a wet paper towel with parafilm on top. 50 μl of smFISH probes diluted 1:100 in hybridization buffer (0.45 μm filtered(Fisher Scientific Co LLC: 09-719D), 10% dextran sulfate(Fisher Scientific Co LLC: S4030), 10% formamide (Fisher Scientific Co LLC: BP227500), 1x nuclease-free SSC(Life Technologies Corporation: 15557044), diluted in nuclease-free water(Fisher Scientific Co LLC: 10977023)) were dropped onto the parafilm. Glass slips were flipped onto the smFISH probes, the hybridization chambers were sealed with parafilm and incubated overnight at 37°C. The next day, slips were washed twice in Buffer A, then once in 2x SSC. Slips were then mounted on slides using Vectashield (Vector Laboratories: 101098–044) and dried. Images were taken on a Nikon Eclipse Ti2 with a CFI60 Plan Apochromat Lambda D 100X Oil Immersion Objective Lens, N.A. 1.45, W.D. 0.13mm, F.O.V. 25mm, DIC, Spring Loaded. The filter set included: C-FL DAPI Filter Set, High-Signal-Noise, Semrock Brightline, Excitation: 356/30nm (341–371nm). The colocalization of ALT with each protein was quantified by estimating the Spearman’s rank correlation coefficient using the Coloc2 plugin of Fiji Image J software. The average values of correlation coefficients from a minimum of three to four images were calculated for each splicing factor.

### RNAscope assay

Coverslips were washed with poly-L-lysine (0.01%) for 15 min and then rinsed 3 times with sterile PBS 3 followed by 3 washes with 100% ethanol for 5 min each. The coverslips were left to air dry for minimum of 2 h. BCBL-1 or iSLK-WT-BAC16 infected cells were seeded overnight on the coated coverslips and were induced with TPA (20 ng/ml) and sodium butyrate (2 mM) or doxycycline (1 μg/ml) and sodium butyrate (1 mM), respectively, at a confluency of 40–50%. Cells were harvested 48 h post-induction. The coverslips were rinsed with PBS once and cells were fixed by incubating with 10% natural-buffered formalin (NBF) for 30 min. After a PBS rinse, the coverslips were washed consecutively in 50%, 75%, and 100% ethanol for 1 min each. After the final wash in 100% ethanol, the coverslips were stored at -20 °C. Coverslips with fixed cells were retrieved from 100% ethanol and immobilized onto a glass slide. A hydrophobic barrier was created around the coverslip. Cells were rehydrated by consecutive washes with 100%, 75%, and 50% ethanol for 1 min followed by 1 wash with PBS. Next, the coverslips were incubated in PBST (PBS with 0.1% Tween 20) for 10 min, washed twice with PBS, and treated with hydrogen peroxide for 10 min. After 2 washes with PBS, cells were incubated in Protease III diluted 1:15 in PBS in a Humidity Control Tray for 10 min. This was followed by 2 final PBS washes. Slides were incubated with RNAscope probes to ALT, designed by ACD Biosciences, targeting the 3’-end of ALT at 40 °C for 2 h and the signal was amplified using the Fluorescence v2 kit according to the manufacturer’s instructions. Staining of the amplified signal with Cyanine3 was monitored using a Zeiss Confocal LSM00 Microscope (University of Florida ICBR Cytometry Core Facility).

### Immunofluorescence Assay

Coverslips were processed for immunofluorescence immediately after the RNAscope. Slides stained with ALT using RNAscope were washed twice in TBST Wash Buffer (500 μL 10% Tween 20 in 1 L of 1X Tris-buffer saline) with gentle agitation for 2 min. The slides were then incubated in 10% normal serum in TBS overnight at 4 °C in a humidity control tray, subsequently followed by primary antibody (PTBP2; Sigma #HPA047420 1:100 and SRSF1; Invitrogen #32–4500 1:250) staining for 1 h and secondary antibody staining (Invitrogen Alexa-Fluor 488) for 30 min at RT. After each antibody incubation, coverslips were washed three times with TBST. After washing with TBST, slides were mounted using Prolong Gold mounting media (Thermo Fisher) and allowed to dry overnight.

### ALT overexpression in uninfected-iSLK cells and RNA sequencing

iSLK cells at 50–60% confluency in 6-well plate were transfected with either 2 μg of pcDNA-ALT or control pcDNA plasmid using X-tremeGENE transfection reagent as per manufacturer’s instructions. The cells were harvested after 48 h and 72 h of transfection and RNA was isolated from all samples using TRIzoL(Invitrogen). Each RNA was treated with DNaseI and re-purified using the Monarch Spin RNA Cleanup Kit (NEB #T2030). RT-qPCR was performed as mentioned above to detect ALT expression. Ribosomal RNA was depleted from DNase treated RNA by using the rRNA depletion kit (NEB #E6310) and Illumina libraries were prepared from 50 ng of rRNA depleted samples, three bioreplicates each of pcDNA-ALT and control transfected (72 h) samples using the NEB Ultra II Directional RNA Library Prep Kit for Illumina (NEB #E7760). Prepared libraries were barcoded using the index primers (NEB #6440). All libraries were loaded in equal concentration to perform paired-end sequencing on NovaSeq X instrument to obtain minimum of 200 million paired-end (150 bp) reads per sample.

### GapmeR transfection for ALT knockdown and efficiency test in BCBL-1 cells

GapmeRs targeting different regions on the ALT lncRNA were designed and purchased from Qiagen (QIAGEN, Valencia, CA) and were transfected into BCBL-1 cells by Neon Transfection System (Invitrogen) as per manufacturer’s protocol. Briefly, 5x10^6^ BCBL-1 cells were resuspended in 100 ul of Buffer T and transfected with 10 nM final concentration of pooled or single gapmeRs with three pulses (1600 V, pulse width 10 mS) and cultured for 24 h before induction as required for the assay. To test the efficiency of ALT knockdown, RNA was isolated from transfected cells after 48 h of induction. 1 μg of RNA from each sample was used to prepare cDNA and detect ALT expression by qRT-PCR as described earlier in this section.

### RNAseq from GapmeR transfected BCBL-1 cells

20 million BCBL-1 cells were transfected in with either control-GapmeRs (Set A) or different set of pooled ALT-GapmeRs including Set B (#1 and #2), Set C (#3 and #4) and Set D (#1, #2, #3 and #4), as mentioned above, in three bioreplicates each. After 24 h of transfection, cells were induced with TPA (20 ng/ml) and sodium butyrate (2 mM) and incubated for another 48 h. RNA isolated from induced transfected cells was treated with Turbo DNAse (Thermo Fisher, AM1907). Ribosomal RNA was depleted from DNase treated RNA by using the rRNA depletion kit (NEB #E6310) and Illumina libraries were prepared from 50 ng of rRNA depleted samples (RIN > 7) using the NEB Ultra II Directional RNA Library Prep Kit for Illumina (NEB #E7760). Prepared libraries were barcoded using the index primers (NEB #6440). All libraries were loaded in equal concentration to perform paired-end sequencing on NovaSeq 6000 instrument to obtain minimum of 60 million paired-end (150 bp) reads per sample.

### SpliceTool analysis

An FDR threshold of 0.05 and rMATS JCEC (Junction Counts and Exon Coverage) files were used for all analyses [24,26]. The human Ensembl 90 annotation was used for splicing event count including SEIntronExonSizes, SENumberSkipped, SETranslateNMD, SEUnannotated, and RIIntronExonSizes (Bioinformatics core, University of Tulane).

### GapmeR transfection for ALT knockdown and efficiency test in iSLK cells

For GapmeR transfection in iSLK-WT-BAC or iSLK-219-BAC-infected cells, we seeded cells at a density of 2.5x10^5^ cells per well in 6 well plate followed by transfection next morning with GapmeRs (40 nM) targeting ALT (pooled GapmeRs #1, #3 and #4) or control GapmeRs. All transfections were done using Lipofectamine RNAi max as per the manufacturer’s protocol. Cells were induced after 24 h of transfection as required for the assay. To test the efficiency of ALT knockdown, RNA was isolated from transfected cells after 48 h of induction. 1 μg of RNA from each sample was used to prepare cDNA and detect ALT expression by qRT-PCR as described earlier in this section.

### PCR validation for skipped exons

iSLK-WT-BAC-infected cells transfected with either GapmeRs targeting ALT (pooled GapmeRs #1, #3 and #4) or control GapmeRs were induced with 1 µg/mL doxycycline and 1mM sodium butyrate after 24 h of transfection. RNA was isolated from these cells after 48 h of induction. Forward and reverse PCR primer were designed in the upstream and the downstream exon of the skipped exon, respectively. The PCR product was run on the agarose gel to detect the amplification of skipped exon of interest, which is the longer PCR product.

### Viral gene expression profile analysis

iSLK-219-BAC-infected cells transfected with either GapmeRs targeting ALT (pooled GapmeRs #1, #3 and #4) or control GapmeRs were induced with 1 µg/mL doxycycline and 1 mM sodium butyrate after 24 h of transfection. RNA was isolated from these cells after 48 h of induction. Forward and reverse PCR primer were designed in the upstream and the downstream exon of the skipped exon, respectively. The PCR product was run on the agarose gel to detect the amplification of skipped exon of interest, which is the longer PCR product.

### Supernatant transfer assay

For supernatant transfer assay, iSLK-219-BAC-infected cells transfected with either GapmeRs targeting ALT (pooled GapmeRs #1, #3 and #4) or control GapmeRs were induced with 1 µg/mL doxycycline and 1mM sodium butyrate after 24 h of transfection. 72 h post induction, the supernatant was collected and filtered through a 0.45 µM syringe filter. The fluorescence intensity of each well was then GFP and RFP fluorescence was scanned and quantified using a spectrophotometer plate reader to generate the RFP/GFP ratio under each condition. Next, 2 ml of the filtered supernatant was added to 293T cells seeded at a density of 2.5x10^5^ cells per well in 6 well plate. 8 µg/ml of polybrene was also added to each well. The plate was centrifuged at 1,500xg for 2 h at 37C. Imaging for GFP positive cells was performed after 24 h of transduction to estimate the infected cells.

## Supporting information

S1 Fig RNAscope of ALT in BCBL-1 cells. The top panel shows the uninduced or latently infected BCBL-1 cells. Middle and the bottom panels show the BCBL-1 cells induced (24 h) in the presence or absence of KSHV replication inhibitor (PAA) representing the early lytic (middle panel) and late lytic (bottom panel) phase of viral replication. Nucleus is stained with DAPI.
(EPS)

S2 Fig Quantification of ALT-MBNL1 co-localization. 2D intensity plots showing the correlation between ALT smFISH and MBNL1 immunofluorescence signals observed by confocal microscopy in **a)** iSLK cells and **b)** BC-3 cells. The average spearman’s rank correlation value of ALT and MBNL1 colocalization was calculated as 0.64 in iSLK cells and 0.54 in BC-3 cells.
(EPS)

S3 Fig ALT RNAscope in BC-3 cells. RNAscope for ALT (orange) combined with immunofluorescence assay for interacting proteins PTBP2 and SRSF1 in 48 h induced BC-3 cells. FITC-conjugated secondary antibodies are used to detect the PTBP2 and SRSF1 (green). Nucleus was stained with DAPI (blue). Bright yellow signal in the merge images show the colocalization of ALT with respective proteins.
(EPS)

S4 Fig Alternative Splicing Changes induced in iSLK cells with ALT expression. **a)** Numbers of alternative splicing events identified by rMATS in the iSLK-WT-BAC16 infected cells after 48 h post induction compared to the latent cells. **b)** Relative expression of ALT in iSLK cells transfected with ALT plasmid with respect to that in iSLK cells transfected with control plasmid after 48 h and 72 h of transfection. **c)** Numbers of alternative splicing events identified by rMATS in the iSLK cells transfected with ALT expression plasmid compared to iSLK cells transfected with control plasmid after 72 h of transfection.
(EPS)

S5 Fig Perturbation of Exon Skipping in ALT dependent manner. **a-b)** An IGV plot depicting the RNAseq reads from latent and reactivated TREx-BCBL1-Rta cells, nanopore long reads from latent and reactivated BCBL-1 cells and RNAseq reads from either control-GapmeR treated or ALT-GapmeR treated BCBL-1 cells, spanning the complete region of RBCK1–213 (a) and NOL8–207 (b) host transcripts. All reactivated and control GapmeR treated samples demonstrate the conditions with high ALT expression (blue), and all latent and ALT-GapmeR treated samples demonstrate the conditions with low ALT (red). The blocks in grey highlight the exons which showed significant change in skipping in all conditions depending on ALT expression.
(EPS)

S6 Fig Effect of ALT knockdown on the induction potential of KSHV in iSLK-WT-BAC cells. Ratio of total infected iSLK.219 cells observed as GFP positive and the fraction of cell that are induced upon lytic reactivation by doxycycline after ALT knockdown or control GapmeR treatment.
(EPS)

S1 Table RAP-enriched-proteins. (XLSX)

S2 Table T-to-C-transition-details-bedgraph. (XLSX)

S3 Table Primers-and-GapmeRs. (XLSX)
